# Hair bundles of cochlear outer hair cells are shaped to minimize their fluid-dynamic resistance

**DOI:** 10.1038/s41598-017-03773-y

**Published:** 2017-06-15

**Authors:** Nikola Ciganović, Amanuel Wolde-Kidan, Tobias Reichenbach

**Affiliations:** 1 Imperial College London, Department of Bioengineering, London, SW7 2AZ United Kingdom; 2 Freie Universität Berlin, Department of Physics, 14195 Berlin, Germany

## Abstract

The mammalian sense of hearing relies on two types of sensory cells: inner hair cells transmit the auditory stimulus to the brain, while outer hair cells mechanically modulate the stimulus through active feedback. Stimulation of a hair cell is mediated by displacements of its mechanosensitive hair bundle which protrudes from the apical surface of the cell into a narrow fluid-filled space between reticular lamina and tectorial membrane. While hair bundles of inner hair cells are of linear shape, those of outer hair cells exhibit a distinctive V-shape. The biophysical rationale behind this morphology, however, remains unknown. Here we use analytical and computational methods to study the fluid flow across rows of differently shaped hair bundles. We find that rows of V-shaped hair bundles have a considerably reduced resistance to crossflow, and that the biologically observed shapes of hair bundles of outer hair cells are near-optimal in this regard. This observation accords with the function of outer hair cells and lends support to the recent hypothesis that inner hair cells are stimulated by a net flow, in addition to the well-established shear flow that arises from shearing between the reticular lamina and the tectorial membrane.

## Introduction

Fluid dynamics plays an important role in many biological systems and the resulting constraints on functioning and efficiency have indeed been shown to be an important factor in evolution^1^. Fungal spores, for instance, are shaped to minimize fluid-dynamic drag^2^. As another example, shark skin reduces hydrodynamic drag through a so-called riblet structure, a principle that is now also used in engineering applications^3^. It is therefore tempting to speculate that drag-related optimization might have shaped structures also in the mammalian inner ear, a highly-evolved sensory organ that employs fluid dynamics.

**Figure 1:**
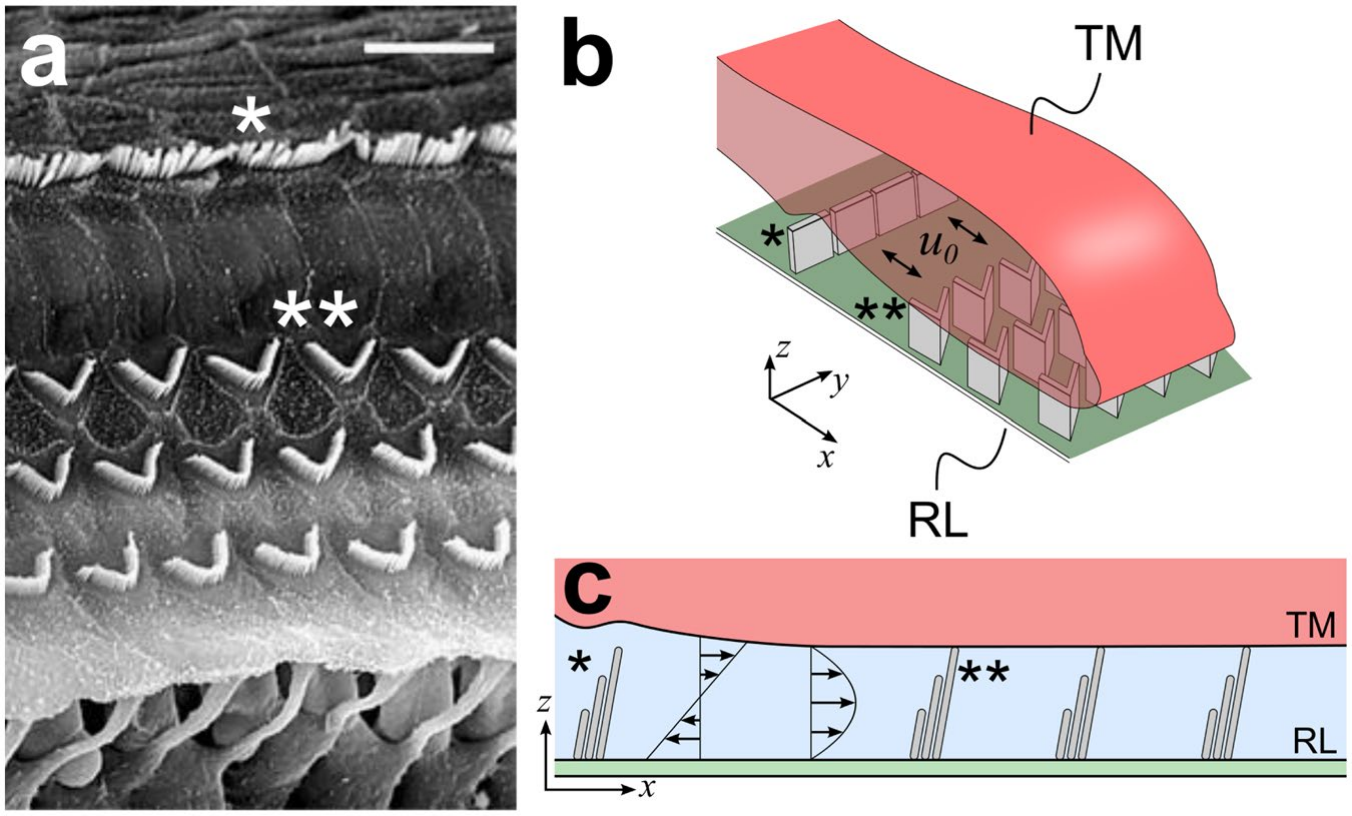
Anatomical environment of cochlear hair bundles. (**a**) Scanning electron microscopy shows hair bundles protruding from the reticular lamina^32^ (scale bar 15 *μ*m, tectorial membrane removed). The hair bundles of inner hair cells are planar (asterisk) whereas those of outer hair cells have a characteristic V-shape (double asterisk). (**b**) Hair bundles of the outer hair cells connect the reticular lamina (RL) to the tectorial membrane (TM). Oscillatory fluid flow occurs in the radial direction, perpendicular to the rows of hair cells (double-sided arrow). (**c**) Fluid flow around hair bundles can include shear flow as well as net flow. The latter may arise from squeezing of the gap between the reticular lamina and the tectorial membrane.

The inner ear transduces mechanical sound vibration into electrical signals through displacement of highly specialized organelles composed of densely bundled stereocilia, the *hair bundles*. Each hair bundle protrudes from the apical surface of a hair cell into a fluid-filled space. In mammals, two types of hair cells are aligned along the length of the cochlea in a regular pattern: typically three rows of *outer hair cells* lie in parallel to one row of *inner hair cells* (Fig. 1). The hair bundles of inner and outer hair cells are easily distinguished by their shape. Stereocilia of inner hair cells are arranged in linear bundles, while hair bundles of outer hair cells exhibit a characteristic V-shape (Fig. 1a,b). These different morphologies likely reflect the distinct functions of inner and outer hair cells. Outer hair cells underlie the cochlear active process: they can amplify small sound vibration and thereby significantly enhance the sensitivity and dynamic range of our hearing^4^. The inner hair cells, in contrast, do not provide amplification but transmit the electrical sound-evoked signals to afferent auditory-nerve fibers^5^.

The hair bundles of outer hair cells connect the reticular lamina, in which the apical surfaces of the hair cells are embedded, to the tectorial membrane that lies in parallel above it (Fig. 1b,c). They are therefore stimulated by the sound-evoked shearing between the reticular lamina and the tectorial membrane which occurs in the radial direction, perpendicular to the rows of hair cells. In contrast, hair bundles of inner hair cells are anchored only in the reticular lamina and not in the tectorial membrane, and are stimulated by radial fluid flow between the two structures. This fluid flow can comprise shear flow, as elicited by shearing between the reticular lamina and the tectorial membrane, as well as a net flow as could result from squeezing of the narrow space between the two structures^6, 7^ (Fig. 1c). Viscous coupling between the reticular lamina and the tectorial membrane likely inhibits the latter component at high frequencies^8–10^. However, recent work has emphasized the potential physiological importance of net flow at low auditory frequencies of up to about 3 kHz that are of particular importance for the perception of speech and music^6, 11, 12^. As the hair bundles of outer hair cells move with the shear between reticular lamina and tectorial membrane, they experience no drag from the resulting shear flow. They will, however, present an obstacle to any net flow and may thus reduce the stimulation of the hair bundles of the inner hair cells. Here we investigate systematically the influence of different shapes of hair bundles of outer hair cells on the bundles’ resistance to such crossflow.

## Results

### Flow properties around cochlear hair bundles

The fluid flow around the hair bundles displays important characteristics that aid its analysis.

First, fluid flow around the hair bundles occurs at low Reynolds numbers, which allows us to linearize the flow equations. Hair bundles and hair cells are indeed microscopic entities. The lattice constant of a row of hair bundles is about *a* = 8 *μ*m^13^. The relevant fluid displacements vary between 0.1–30 nm and occur at frequencies of 10 Hz–100 kHz, resulting in velocities of the radial shear flow between 10 nm·s^−1^ and 10 mm·s^−1^ ^14^. The radial net flow may exceed the radial shear flow, but experimental investigations of the nanomechanics of the subtectorial space show that it is likely not more than tenfold larger^11^. The velocity amplitude *u*_0_ is thefore limited to values of 10 nm·s^−1^ to 0.1 m·s^−1^. The fluid around the hair bundles, endolymph, is similar to water in its mechanical properties. Hence, the density is *ρ* = 1,000 kg·m^−3^ and the viscosity is *μ* = 1 mPa·s. This yields Reynolds numbers *Re* = *u*_0_ *aρ*/*μ* between 10^−8^ and 10^−1^, much below one.

Second, the Womersley parameter $\alpha =a\sqrt{2\pi f\rho /\mu }$ is small with a value below one for frequencies less than 6 kHz. Subtectorial net flow is likely to be physiologically relevant mainly for the comparatively low frequencies below 3 kHz^11^ which include the frequency range that is mostly relevant for human speech^15^. We can thus neglect the oscillatory nature of the fluid flow and assume it to be quasi-steady^16, 17^. The fluid motion is then adequately characterized by Stokes flow.

Third, flow between the densely spaced individual stereocilia that constitute a hair bundle is highly suppressed for audible frequencies. Below 100 Hz, top connectors between stereocilia ensure that the bundle moves coherently, whereas above 100 Hz viscous forces make the bundle move as a unit^18^. We therefore represent the hair bundles as impermeable in our analysis. Furthermore, a hair bundle’s stereocilia are sufficiently stiff such that the bundle can be modeled as rigid when analyzing the surrounding flow^17^.

Fourth, the flow is confined to the thin space between the parallel reticular lamina and tectorial membrane, and occurs in parallel to these structures (Fig. 1a,b). To a first approximation, we can therefore analyze the fluid dynamics in a two-dimensional layer at a particular height between reticular lamina and tectorial membrane. Moreover, the effects of multiple parallel hair-bundle rows on the fluid flow can be expected to be approximately additive, as the flow surrounding the bundles is approximately linear. It hence suffices to consider a single row of hair bundles only in our analysis.

Finally, only the radial component of the flow which is directed perpendicularly to the inner hair bundles contributes to their stimulation and is therefore of physiological relevance. Although acoustically-evoked flow in the cochlea may include a longitudinal component as well, we can therefore focus on radial flow only^19, 20^.

### Drag reduction by V-shaped hair bundles

#### Analytical results

We first seek to gain analytical understanding of the characteristics of the fluid flow across a row of hair bundles.

**Figure 2:**
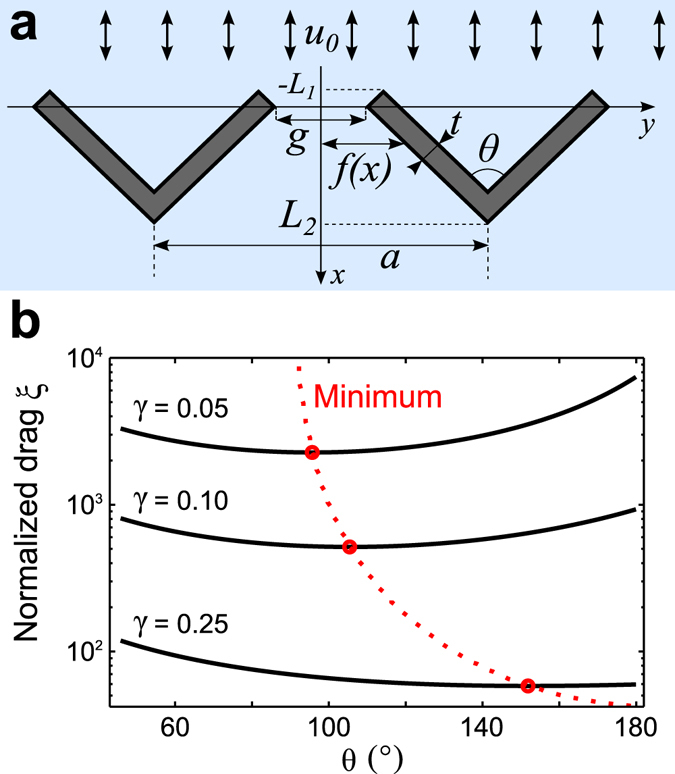
Analytical approximation of the normalized drag of a hair bundle in a row. (**a**) Schematic of the analyzed geometry. See text and *Methods* for parameters. (**b**) The drag is minimal at an optimal angle θ* (red) of the hair-bundle shape. The optimal angle shifts to larger values for increasing gap size.

We adapt a method by Keller who analyzed Stokes flow past a dense grating of identical cylinders^21^. Consider a regular row of hair bundles that extends longitudinally along the *y*-axis (Fig. 2a). The radial flow occurs parallel to the *x*-axis which we choose to lie midway between two neighboring bundles. We want to estimate the drag on a hair bundle as a function of the geometrical parameters: the lattice constant *a*, the angle of the hair bundle’s shape *θ*, the gap *g* between two neighboring bundles, and the hair-bundle thickness *t*.

The drag force *F*_*D*_ per unit height on a bundle is proportional to the pressure drop Δ*p* across the bundle, *F*_*D*_ = *a*Δ*p*. We expect the pressure drop to occur predominantly in the narrow gap between two bundles, so that in the limit of a small gap size *g* lubrication theory can be applied^21^. Since in the regime of Stokes flow the drag force is proportional to both the fluid velocity *u*_0_ and the fluid viscosity *μ*, we characterize the bundle’s drag by the normalized drag *ξ* = *F*_*D*_/(*μu*_0_). Note that *ξ* is dimensionless since the force per unit height *F*_*D*_ has the units N·m^−1^. Introducing the dimensionless gap parameter *γ* = *g*/*a* and the thickness parameter *τ* = *t*/*a*, the normalized drag *ξ* is derived as

1 $$\xi \approx \frac{3}{{\gamma }^{2}}[\frac{1}{\tan (\theta /\mathrm{2)}}(1-{\gamma }^{2})+\,\tan (\theta /\mathrm{2)}(1-{[1+\frac{2\tau }{\gamma }\cos (\theta /\mathrm{2)}]}^{-2})].$$

(see *Methods* for details of the calculation).

Because hair bundles with angles below around 40° would no longer fit onto the surface of a single hair cell, we need only consider angles between 40° and 180° (Fig. 2b). We note the following qualitative features of the analytical approximation (1).

First, given a fixed gap size, the drag exhibits a minimum at an optimal angle θ* of the hair bundle’s shape.

Second, the hair bundle’s angle with minimal fluid-dynamic resistance depends on the gap size. It tends to 90° for diminishing gap (*γ* → 0) and shifts towards larger angles as the gap is increased. However, we note that our analytical approximation has been derived for the case of small gap sizes and loses validity otherwise.

Third, in the limit of a vanishing gap size (*γ* → 0), the drag increases in proportion to *γ* ^−2^.

Fourth, varying the gap parameter changes the dependence of the drag on the hair bundle’s angle. For small gap sizes, the highest drag occurs for *θ* = 180° and is substantially higher than the lowest achievable drag at the optimal angle θ*. For larger gap parameters, the highest drag values shift towards smaller angles, and the reduction in drag of the optimal shape as compared to flat bundles such as those of inner hair cells becomes less pronounced.

These results are in striking agreement with the shape and function of the hair bundles of inner and outer hair cells: many outer hair bundles have angles of about 90°, thus minimizing their resistance to fluid flow^20^. In contrast, inner hair cells have closely spaced planar hair bundles that maximize drag, and thus also their sensitivity to radial fluid velocity.

#### Computational results

**Figure 3:**
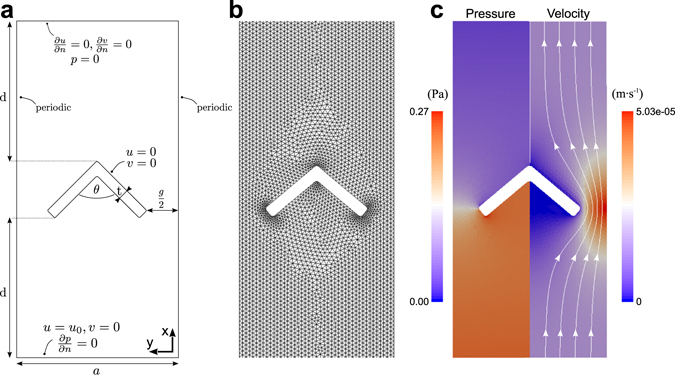
Computational analysis of the flow across hair bundles. (**a**) Geometry of the computational domain and boundary conditions. (**b**) Triangular discretization of the computational domain. (**c**) Exemplary computationally obtained solution for the velocity and pressure fields. Here the tip angle is *θ* = 100°, the gap parameter is *γ* = 0.33, and the height of the bundle is 4 *μ*m. The slice shown is at mid-height of the hair bundle.

For a more detailed analysis involving the full Navier-Stokes equations, we carried out computational fluid dynamics (CFD) simulations using the open-source CFD-code OpenFOAM^22, 23^. As computational domain we chose one unit cell of a row of hair bundles, containing one hair bundle (Fig. 3). We imposed periodic boundary conditions on the lateral sides to simulate an infinite row of hair bundles in the longitudinal *y*-direction (Fig. 3a). Fluid enters along the radial *x*-direction at velocity *u*_0_, either at the boundary facing the hair bundle’s tip or away from it, and exits at the opposite boundary where a fixed reference pressure is set at *p* = 0. We chose the domain large enough along the *x*-axis so that boundary effects become negligible in the region immediately surrounding the hair bundle; away from the hair bundle, the resulting flows were approximately uniform (Fig. 3c). The bundle is represented as an impermeable wall at which a no-slip condition applies. We simulated the flow for different angles *θ* of the hair bundle’s shape as well as for different values of the gap parameter *γ* while keeping the hair bundle spacing *a* and thickness *t* constant. Indeed, the latter parameters show no significant variation between different hair-cell rows or along the cochlea^20^.

We performed flow simulations in both two and three dimensions. The 2D-simulations correspond best to the analytical approximation described above. However, they neglect the finite height of the narrow space between reticular lamina and tectorial membrane, and correspond to a space of infinite height. The finite height of this space is modelled in our 3D-simulations, where the hair bundle is attached at its base and its apex to parallel no-slip surfaces. We simulated hair bundles with heights *h* = 2 *μ*m and *h* = 4 *μ*m in order to assess the dependence of the results on the height of the fluid space.

**Figure 4:**
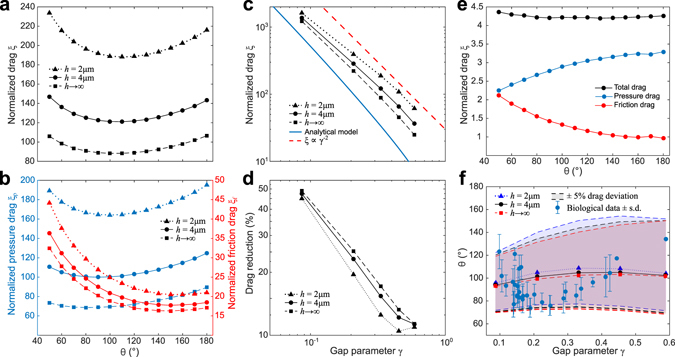
Dependence of the drag on hair-bundle shape for flow across a row of hair bundles. Results are shown from 3D simulations with short (*h* = 2 *μ*m, triangles) and tall (*h* = 4 *μ*m, circles) hair bundles, as well as from 2D simulations which are equivalent to infinitely tall hair bundles (squares). (**a**) The normalized drag for a given gap parameter (here *γ* = 0.33) is minimal for a hair-bundle shape with tip angle ${\theta }_{{\rm{num}}}^{\ast }\approx {100}^{\circ }$. (**b**) The minimal drag at the optimal angle emerges from a trade-off between friction drag (red) and pressure drag (blue). (**c**) The drag increases with decreasing gap parameter *γ* approximately as *γ* ^−2^ for a hair bundle with an angle $\theta ={100}^{\circ }\approx {\theta }_{{\rm{num}}}^{\ast }$. (**d**) The reduction in drag at the optimal angle compared to the case *θ* = 180° is larger for smaller gap sizes. (**e**) The total drag on a single hair bundle in unbounded flow shows little dependence on the shape of the bundle and varies by only about 3.7%. Significant drag reduction for V-shaped hair bundles as shown in panels a and d is therefore particular to dense rows of hair bundles as found in the cochlea. (**f**) The numerically-computed optimal angle of the hair bundle’s shape varies slightly with gap sizes (blue, black, and red lines with markers), and so does the range of angles for which the drag is at most 5% higher than at the optimal angle (shading in corresponding colors). All biologically-observed angles of outer hair cells’ hair bundles fall into this range.

The dependence of the simulated drag on the hair bundle’s angle *θ* for a wide range of gap parameters *γ* exhibits a profile very similar to the one found in our analytical approximation (Fig. 4a). In particular, the minimal drag occurs close to an angle of 100°. Similar results are obtained also for higher Reynolds numbers (up to *Re* = 0.8) and oscillatory flow with frequencies of up to 100 kHz. As expected for low Reynolds number flow, the dependence of the drag on the direction of the flow is negligible.

The simulations allowed us to separate the total drag into pressure and friction drag. The pressure drag denotes the net pressure that acts on the hair bundle, whereas the friction drag arises from viscous shear stresses along the bundle’s surface. Analyzing the dependence of pressure and friction drag on the hair bundle’s angle *θ* shows that the minimal net drag at an optimal angle results from an interplay of the two components (Fig. 4b). The friction drag is high for acute angles and decreases towards more planar hair-bundle shapes. This is partly due to the fact that, as we keep the gap parameter *γ* constant, the surface area of the hair bundle facing the flow decreases for larger angles. Furthermore, our simulations show that for a wide range of angles the flow in the space between two bundles is radiating from or towards a point near the intersection of the imagined extensions of the legs of two neighboring bundles (see also Fig. 3c). In two dimensions, such flow can be approximated by the Jeffery-Hamel solution for viscous flow in a wedge with a source or sink in its tip^16^. This theory predicts higher shear stresses at the walls for more acute angles of the wedge which contributes to the increase of friction drag. The pressure drag, on the other hand, increases for more planar shapes of the hair bundles (Fig. 4b). Because both pressure and friction drag contribute to a similar degree to the net drag, the latter exhibits a minimum at an optimal value of the hair bundle’s angle of around ${\theta }_{{\rm{num}}}^{\ast }\approx {100}^{\circ }$ where both drag components are comparatively small (Fig. 4a).

Consistent with our analytical approximation (1), the drag increases for smaller gap parameters *γ* (Fig. 4c) approximately as *γ* ^−2^, which is in agreement with previous results on the drag of a regular grid of circular cylinders as well^24^. This scaling is particularly apparent for the 2D-simulations, as should be expected.

The results show that while the drag coefficient depends on the distance between the reticular lamina and the tectorial membrane, the shape of the drag’s dependence on the hair bundle angle *θ*, and the minimum around ${\theta }_{{\rm{num}}}^{\ast }\approx {100}^{\circ }$, remain unaffected (Fig. 4a).

To quantify the reduction in drag at the optimal hair-bundle angle we fitted a fourth-order polynomial to the drag’s dependence on the angle *θ*, from which we determined the optimal angle ${\theta }_{{\rm{num}}}^{\ast }$ as well as the drag at that configuration. We then compared this minimal drag to the drag of a linear hair bundle (*θ* = 180°). As in the analytical model, the proportional reduction in drag is about 50% for the smallest gap parameter *γ* = 0.08 found in the mammalian inner ear, and decreases for larger values (Fig. 4d). Hair-bundle shape hence becomes more critical with regard to drag for smaller gap sizes.

Hair bundles of outer hair cells can be shaped as a W rather than a V, with a small indentation at the tip. Because our analytical calculation shows that the drag results from the gap between the hair bundles, the precise shape of the hair bundles’ tips should be unimportant. To verify this numerically, we performed simulations with a row of hair bundles that had an indentation of 0.1 *μ*m at the tip. We obtained a drag that was virtually the same as that of a row of V-shaped bundles: the drag of both structures differed by less than 0.002%.

Finally, to investigate the influence of the arrangement of hair bundles in rows, we simulated the unbounded flow field around a single hair bundle, with no neighboring hair bundles. Its total drag shows only little dependence on the angle *θ* of the hair bundle’s shape (Fig. 4e). The distinct minimum in drag at an angle of about 100° observed earlier, as well as the sizable drag reduction there for small gap sizes, are hence a consequence of the arrangement of many hair bundles in dense rows.

### Biological hair-bundle shapes are near-optimal

Data on geometric parameters of hair bundles of outer hair cells in guinea pig have been collected from different locations along the cochlea by Yarin *et al*.^20^. These data are, to the best of our knowledge, the only source of systematic information on cochlear hair-bundle shapes. Although the data suffer from shrinkage of around 50% due to the tissue preparation, the shrinkage is probably homogeneous so that the hair bundle opening angles are represented accurately^20, 25^.

The observed opening angles of hair bundles lie between 75° and 135° and vary in a seemingly systematic fashion with gap size (Fig. 4f). In particular, opening angles between 100° and 120° occur for small gap parameters of about 0.1. The angles drop to around 80° for somehwat larger gap parameters around 0.2 and then increase again to values between 100° and 120° for larger gaps.

While the particular dependence of the opening angles on the gap size is not predicted by our model, the overall range of opening angles is (Fig. 4f). Indeed, if we consider the angular range in which the drag varies by no more than 5% from its minimal value in our simulations, we find that all biologically-observed hair-bundle shapes fall into this range, between approximately 70° and 135°. The biological data cluster particularly around the predicted optimal angle for smaller values of the gap size where the 5% drag deviation corresponds to a smaller angular range.

## Discussion

We have found that hair bundles of cochlear outer hair cells are optimized for hydrodynamic drag: through their V shape they significantly reduce their resistance to crossflow. Previous experimental work by Frommer and Steele^26^ and computational work by Baumgart and coworkers^17^ has already suggested that rows of V-shaped hair bundles have a reduced fluid-dynamic resistance as compared to those with linear hair bundles. However, a systematic study of hair-bundle shapes and their effect on surrounding fluid flow has, to the best of our knowledge, not been undertaken before. We have shown here that the drag of biologically-observed shapes of hair bundles of outer hair bundles deviates, in fact, only little from the minimal theoretically achievable drag, suggesting that reducing the drag of hair-bundle rows is part of the underlying design principle.

The drag reduction accords with the physiological function of outer hair cells: their hair bundles are not stimulated by fluid flow, but by shearing between the reticular lamina and the tectorial membrane. Through active feedback they amplify the motion of the reticular lamina which potentially elicits a crossflow that then stimulates the hair bundles of the inner hair cells, at least for frequencies below 3 kHz^6, 11, 12, 27^. Minimizing their resistance to crossflow can therefore help the outer hair cells to elicit a large crossflow from the active deformation of the organ of Corti. Furthermore, the drag reduction found here could also facilitate active hair-bundle motion against the surrounding fluid: active hair bundle forces can potentially lead to larger hair-bundle vibration as the bundle’s drag is reduced through its V shape.

The hair bundles of the inner hair cells are not connected to the tectorial membrane, and are therefore not stimulated directly by shearing between the reticular lamina and the tectorial membrane. Instead, both shear flow and crossflow between the two structures displace these hair bundles, and we therefore expect them to maximize resistance to such fluid flow. Our analytical and numerical computations suggest that this is indeed the case: at a small gap size, as found for the hair bundles of inner hair cells, their planar geometry maximizes the hydrodynamic drag (Figs 2b and 4a). However, these results have been obtained for hair bundles that are anchored in the tectorial membrane. In reality, fluid flow may occur in the space between the tips of the inner hair cell stereocilia and the tectorial membrane and may be further influenced by the presence of a bulge running along the tectorial membrane, the so-called Hensen stripe^28, 29^.

The shape of a single particle in Stokes flow has a rather small effect on the drag the particle experiences. For example, for a given particle volume, the drag experienced by a spherical particle in Stokes flow differs by less than 5% from the minimal achievable drag on a particle of optimal, distinctly non-spherical shape^30^. However, the situation is different for cochlear hair bundles. Significant drag reduction through an appropriate morphology becomes possible due to the dense arrangement of hair bundles of multiple cells in a row. The drag-reduction effect reported here is therefore not a property of a single hair bundle, but rather a strategy of the entire hair-bundle row to reduce its resistance to crossflow. In contrast, the presence of multiple parallel rows of hair bundles of outer hair cells does not qualitatively modify the effect. Indeed, due to the linearity of the flow equations, several rows contribute approximately additively to the total drag^21^. Moreover, our simulations show that the flow in front and behind a row of hair bundles only deviates significantly from a uniform flow close to the bundles. Hence, subsequent hair-bundle rows would experience approximately the same flow locally and therefore contribute a similar amount of drag per bundle as is found for a single row, independent of how the different rows are aligned relative to each other.

The analytical approximation that we have developed considers flow in a two-dimensional plane only and relies on the assumption of a linear and quasi-steady flow, which holds for frequencies up to a few kHz. However, our computational fluid dynamics simulation accounts for the full nonlinear Navier-Stokes equation, as well as for the three-dimensional geometry including appropriate boundary conditions at the reticular lamina and the tectorial membrane. The simulation shows that the obtained results hold for the three-dimensional bundle as well as for frequencies of at least up to 100 kHz. Drag optimization by the hair bundles of outer hair cells to subtectorial net flow thus occurs at all audible frequencies, although the net flow may only be physiologically relevant at frequencies below 3 kHz^11^.

The optimal angle that we have computed varies only little with the gap size between neighboring hair bundles (Fig. 4f). The biologically-observed data show, however, a non-monotonic variation: both larger and smaller opening angles are found for small gap sizes, and larger angles occur at larger gaps. Indeed, the smallest angles occur in the midregion of the cochlea, whereas the gap size increases from base to apex. Other factors in addition to drag reduction therefore presumably play a role in determining the opening angle. Further constraints may involve the number of stereocilia necessary to achieve mechanotransduction currents of a sufficient magnitude coupled with space constraints, as well as the resulting mechanical properties of the bundles. Nevertheless, since the observed hair bundle shapes all fall in the range of at most 5% deviation from the minimal drag, such additional factors do likely not involve fluid-dynamic constraints; the latter appear crucial in defining a critical range of opening angles in which additional factors can then yield further variation.

## Methods

### Analytical methods

We use lubrication theory to analyze flow between two closely spaced neighboring hair bundles. In this approximation the Stokes equation reduces to

2 $$\frac{\partial p}{\partial x}=\mu \frac{{\partial }^{2}u}{\partial {y}^{2}},$$

where *u* is the *x*-component of the velocity field. Equation (2) can be integrated to

3 $$u=\frac{{y}^{2}-f{(x)}^{2}}{2\mu }\frac{{\rm{d}}p}{{\rm{d}}x},$$

where *f*(*x*) describes the shape of the bundles (Fig. 2a). Due to mass conservation, the flux *q* through any longitudinal surface given by a constant value of *x* between two neighboring hair bundles must be conserved and is given by

4 $$q={\int }_{-f(x)}^{f(x)}u\,{\rm{d}}y=-\frac{2f{(x)}^{3}}{3\mu }\frac{{\rm{d}}p}{{\rm{d}}x}.$$

Since *q* = *au*_0_, we obtain

5 $$\frac{{\rm{d}}p}{{\rm{d}}x}=-\frac{3\mu a{u}_{0}}{2f{(x)}^{3}}\mathrm{.}$$

The pressure drop between two locations *x*_1_ and *x*_2_ follows as

6 $$p({x}_{1})-p({x}_{2})=\frac{3\mu a{u}_{0}}{2}{\int }_{{x}_{1}}^{{x}_{2}}\frac{{\rm{d}}x}{f{(x)}^{3}}.$$

The drag force on a hair bundle per unit height is then *F*_*D*_ ≈ *a*[*p*(−*L*_1_) − *p*(*L*_2_)] where the range from *x* = −*L*_1_ to *x* = *L*_2_ corresponds to the extent of the hair bundle in the radial direction (Fig. 2a). Since in the regime of Stokes flow the drag force *F*_*D*_ is proportional to both the fluid velocity *u*_0_ and the fluid viscosity *μ*, we characterize the bundle’s drag by the normalized drag *ξ* = *F*_*D*_/(*μu*_0_).

For simplicity, we model the shape function *f*(*x*) as a piece-wise linear function,

7 $$f(x)=\{\begin{array}{ll}-\frac{x}{tan(\theta /\mathrm{2)}}+g/\mathrm{2,} & -{L}_{1}\le x\le \mathrm{0,}\\ tan(\theta /\mathrm{2)}x+g/\mathrm{2,} & 0\le x\le {L}_{2},\end{array}$$

thus rendering the evaluation of the integral in equation (6) more tractable. The normalized drag then follows as

8 $$\xi \approx \frac{3}{{\gamma }^{2}}[\frac{1}{\tan (\theta /\mathrm{2)}}(1-{\gamma }^{2})+\,\tan (\theta /\mathrm{2)}(1-{[1+\frac{2\tau }{\gamma }\cos (\theta /\mathrm{2)}]}^{-2})],$$

where we have introduced the dimensionless gap parameter *γ* = *g*/*a* and the thickness parameter *τ* = *t*/*a*.

### Computational methods

Pre-processing for our CFD simulations was accomplished with the open-source software SALOME 7.7.1 (Open Cascade, EDF, CEA). We modeled a single unit cell of a row of hair bundles (Fig. 3a).

The edges of the bundle were rounded with a radius of *r* = 0.1 *μ*m, corresponding to the average radius of a single stereocilium inside a hair bundle^20^. The thickness of the bundle *t* was chosen to represent the average value along the height of the hair bundle and was consequently set to *t* = 0.45 *μ*m^20^. The width of the computational domain *a* in Fig. 3a represents the spacing of the single bundles in the row and was set to *a* = 5.8 *μ*m in accordance with experimental data from^20^. These data were obtained from preparations that contained some shrinkage of the tissues, and the lattice constant is accordingly smaller than the 8–10 *μ*m suggested by typical outer hair cell diameters^13^. However, at the small Reynolds numbers relevant here, the fluid dynamics depends mainly on relative scales and not on absolute values. The radial extent *d* of the domain around the hair bundle, perpendicular to the direction of the row, was chosen large enough so that boundary effects from inlet or outlet in the vicinity of the bundle become negligible (*d* = 10 *μ*m).

Periodic boundary conditions were applied at the lateral sides of the computational domain to simulate an infinite row of hair bundles (Fig. 3a). A non-slip condition was imposed at the surface of the bundle. For the inlet, at one of the radial sides, the velocity *u*_0_ was prescribed, together with a zero-gradient condition for the pressure. At the outlet, that is, at the opposite radial side, the pressure was set to zero (since only differences in pressure are of interest) and the normal gradient of the velocity was set to vanish. The locations of the inlet and outlet were determined based on the direction of flow under investigation.

For the simulation of a solitary hair bundle we modeled only half of the bundle and applied a symmetric boundary condition at the symmetry side of the bundle (passing through the bundle’s midpoint). At the opposite side of the computational domain, a zero normal gradient condition was applied for both velocity and pressure. The radial and lateral extensions of the computational domain had to be larger than for the simulation of a row of bundles to avoid boundary effects. We employed a radial extension of 40 *μ*m and a lateral extension of 30 *μ*m. All other boundary conditions were the same as above.

A triangular mesh was generated for the modeled geometry using the NETGEN algorithm (Fig. 3b)^31^. The discretized Navier-Stokes equations were solved on this mesh using the pimpleFoam-solver of OpenFOAM (Version 2.3.0)^22, 23^.

The discretization-independence of the numerical solution was confirmed by comparison with results with increased mesh resolution.
